# The impact of COVID-19 on the birth rate in Nigeria: a report from population-based registries

**DOI:** 10.53388/idr2023004

**Published:** 2023-02-15

**Authors:** Charlotte Blanche Oguejiofor, Kenechi Miracle Ebubechukwu, George Uchenna Eleje, Emmanuel Onyebuchi Ugwu, Joseph Tochukwu Enebe, Kingsley Emeka Ekwuazi, Chukwuemeka Chukwubuikem Okoro, Boniface Chukwuneme Okpala, Charles Chukwunomunso Okafor, Nnanyelugo Chima Ezeora, Emeka Ifeanyi Iloghalu, Chidebe Christian Anikwe, Chigozie Geoffrey Okafor, Polycarp Uchenna Agu, Emeka Philip Igbodike, Iffiyeosuo Dennis Ake, Arinze Anthony Onwuegbuna, Osita Samuel Umeononihu, Onyedika Promise Anaedu, Odigonma Zinobia Ikpeze, David Chibuike Ikwuka, Henry Ifeanyi Nwaolisa, Ekene Agatha Emeka, Jude Ogechukwu Okoye, Ihechinyerem Kelechi Osuagwu, Angela Ogechukwu Ugwu, Toochukwu Benjamin Ejikeme, Eziamaka Pauline Ezenkwele, Chijioke Ogomegbunam Ezeigwe, Malarchy Ekwunife Nwankwo, Gerald Okanandu Udigwe, Joseph Ifeanyichukwu Ikechebelu, Grace Agbaeze, Chukwuebuka Divine Nwanja, Ahizechukwu Chigoziem Eke

**Affiliations:** 1 Department of Obstetrics and Gynecology, Nnamdi Azikiwe University Teaching Hospital, Nnewi PMB 5025, Nigeria.; 2 Preventive Medicine and Research Department, Clina-Lancet Laboratories, Victoria Island, Lagos 101241, Nigeria.; 3 Effective Care Research Unit, Department of Obstetrics and Gynecology, Nnamdi Azikiwe University, Nnewi Campus 435001, Nigeria.; 4 Department of Obstetrics and Gynecology, College of Medicine, University of Nigeria Enugu Campus, Enugu, Enugu State 400102, Nigeria.; 5 Department of Obstetrics and Gynecology, University of Nigeria Teaching Hospital, Ituku Ozalla, Enugu State 400102, Nigeria.; 6 Department of Obstetrics and Gynecology, ESUT Teaching Hospital, Parklane, Enugu 400102, Nigeria.; 7 Medical Department, Divine Medical Centre, South West Ikoyi, Lagos 1001233, Nigeria.; 8 Department of Obstetrics and Gynecology, Havana Specialist Hospital, Surulere, Lagos 100011, Nigeria.; 9 Clinical Trial Division, Drug Evaluation and Research Directorate, NAFDAC office Complex, Lagos 100011, Nigeria.; 10 Department of Ophthalmology, Nnamdi Azikiwe University, Nnewi campus 435001, Nigeria.; 11 Department of Human Physiology, Nnamdi Azikiwe University Nnewi, Anambra State 435001, Nigeria.; 12 Department of Family Medicine, Faculty of Medicine, Nnamdi Azikiwe University, Awka 435001, Nigeria.; 13 Department of Medical Laboratory Science, Faculty of Health Sciences and Technology, Nnamdi Azikiwe University, Awka 435001, Nigeria.; 14 Department of Health Services, Federal University of Technology, Owerri 460103, Nigeria.; 15 Department of Haematology and Immunology, College of Medicine, University of Nigeria Enugu Campus, Enugu, Enugu State 400102, Nigeria.; 16 Musgrove Park Hospital Somerset NHS, Foundation Trust, United Kingdom.; 17 TLTp Medical (UHS), Southampton WS15, United Kingdom.; 18 Division of Maternal Fetal Medicine, Department of Gynecology and Obstetrics, Johns Hopkins University School of Medicine, Baltimore 21201, U.S.A.

**Keywords:** birth rates, COVID-19, lockdown, Nigeria, pandemic, pre-COVID

## Abstract

**Background and objectives:**

Coronavirus disease 2019 (COVID-19) is a pandemic that has become a major source of morbidity and mortality worldwide, affecting the physical and mental health of individuals influencing reproduction. Despite the threat, it poses to maternal health in sub-Saharan Africa and Nigeria, there is little or no data on the impact it has on fertility, conception, gestation and birth. To compare the birth rate between pre-COVID and COVID times using selected months of the year.

**Materials and methods:**

This was a secondary analysis of cross-sectional analytical study data from the birth registries of three tertiary hospitals, comparing two years [2019 (Pre-COVID)] versus [2020 (COVID era)] using three months of the year (October to December). The data relied upon was obtained from birth registries in three busy maternity clinics all within tertiary hospitals in South-East Nigeria and we aimed at discussing the potential impacts of COVID-19 on fertility in Nigeria. The secondary outcome measures were; mode of delivery, booking status of the participants, maternal age and occupation.

**Results:**

There was a significant decrease in tertiary-hospital based birth rate by 92 births (*P* = 0.0009; 95% CI: −16.0519 to −4.1481) among mothers in all the three hospitals in 2020 during the COVID period (post lockdown months) of October to December. There was a significant difference in the mode of delivery for mothers (*P* = 0.0096) with a 95% confidence interval of 1.0664 to 1.5916, as more gave birth through vaginal delivery during the 2020 COVID-19 period than pre-COVID-19.

**Conclusion:**

Tertiary-hospital based birth rates were reduced during the pandemic. Our multi-centre study extrapolated on possible factors that may have played a role in this decline in their birth rate, which includes but is not limited to; decreased access to hospital care due to the total lockdowns/curfews and worsening inflation and economic recession in the country.

## Introduction

The coronavirus disease 2019 (COVID-19) is a disease caused by SARS-CoV-2 an infectious respiratory virus. It was first reported in Wuhan, China in 2019 and has since then spread to over 213 countries, with up to 5,934,936 cases globally as of 31st of May 2021 [1].

Sneezing, talking and coughing produce respiratory droplets, which are media COVID-19 viruses that can spread between different people in close physical contact. When people fail to wash or sanitize their hands after touching contaminated surfaces, there is a high possibility of spreading as they eventually touch their eyes, faces and nose with those hands [1, 2]. After the first case of COVID-19 was diagnosed in Nigeria on the 27th of February 2020, there have been several lock-downs and policies to curb the spread.

Prior to the onset of COVID-19 vaccinations, the pandemic led to some bad outcomes on socio-economic and mental health invariably causing a negative influence on contraception, fertility, birth and gestation, this can be explained further by remembering that more than half of the global populace were on lockdowns that lasted from weeks to months initially before other measures were put in place and these lockdowns impinged on people’s mental health, their sources of income, their physical, as well as their reproductive health [3].

Nigeria is already battling unemployment and economic recession, which when compounded with the social and mental effects of the pandemic and may result in further influence on fertility, conception and birth. This research aims at discovering what impact the virus had in the past one-year filled with lockdowns. In addition, how factors such as the rise in the unemployment rate, financial insecurity, initial unknown fatality rate associated with SARS-CoV-2 infection for the first half of the year, the initial lack of effective treatments and vaccination, reduction in hospital visits and healthcare services due to the fear of being infected with the virus by individuals, may have impacted on fertility within tertiary hospitals.

A polarized opinion suggested that the lockdowns in several countries may have led partners to comfort each other with regular coitus which can lead to an increase in birth rate, this however was not so as several countries have reported a decline in their birth rate. There has been a gradual decline in births in Italy for over many years, regardless of the pandemics’ input which cannot be measured. In Northern Italy for instance the Metropolitan City of Genoa, from November 2019 to January 2020, there were 875 births, which fell by 12 percent in 2021 resulting in just 770 births [4].

An early look at the impact of COVID-19 in certain states in the USA, specifically California indicated a fall in the birth rate in the last month of 2020 when compared to the previous year’s December [4], this was pointed out by Philip Cohen a sociologist with University of Maryland who also revealed a drop in other states across the country ranging from five to ten percent decrease, there was the unavailability of data from National data of USA fertility as of when a study was conducted [4]. Likewise, in Spain, a preceding report on the effect on fertility of COVID-19 revealed that there was a 23 percent fall in the birth rate in 2020/2021 for the months December and January compared to the same months in 2019/2020 [4].

How sustainable is this decline, the influenza pandemic of 1918 came with an initial decline in birth rate then a baby bust, is this pattern likely to repeat with this COVID pandemic? Keeping in mind that the economic recession in 2008 that led to a decrease in birth rate has not picked up from its pre-2008 rates since the fall [5]. This study compared the birth rate between pre-COVID and COVID times using selected months of the year from the birth registries of the maternity clinics in three tertiary hospitals in South-East Nigeria, discussing the potential impacts of COVID-19 on fertility in Nigeria.

## Materials and methods

This was a secondary analysis of cross-sectional analytical study data from the birth registries of three tertiary hospitals, comparing two years [2019 (Pre-COVID)] versus [2020 (COVID era)] using three months of the year (October to December) [6]. The Tertiary Hospitals selected; the University of Nigeria Teaching Hospital (UNTH) and Enugu State University Teaching Hospital (ESUTH) and Nnamdi Azikiwe University Teaching Hospital (NAUTH) are in the south-eastern part of Nigeria catering for approximately 1 million people within their various cities and other patients from neighboring towns referred to their facility. These hospitals currently see between 5,000 and 10,000 deliveries per year and are often referred to as ‘baby factories. They are funded by the federal and state government and serve as excellent referral facilities for hospitals and maternity centers seeking extensive maternal and child care management.

We analyzed data from these tertiary hospitals. A structured proforma was used to collect socio-demographic and clinical data of the subjects from their National health management information system, daily birth and delivery registries, comparing 2 years (October to December 2019; pre-COVID-19 era) and (October to December 2020; COVID-19 era). Data is reported based on the mode of delivery, age group of mothers, their occupation, and booking status (booked/un-booked).

All quality control checks and duplication checks were performed by the individual registries. We checked for duplication of cases across registry databases. Excel Spreadsheet version 2019 was used for data entry and exported to Statistical Package for Social Sciences version 25 (IBM, Armonk, NY, USA) for analysis. We exported the data to Microsoft Excel for analysis which was later imported to IBM SPSS software. We performed statistical analyses with IBM SPSS version 25 for Windows. For categorical variables, outcomes between the groups were compared using Pearson’s chi-squared tests, and when appropriate, Fisher’s exact tests.

We calculated the odds ratio, 95% confidence intervals, standard deviations and *P*-values for pre-COVID and COVID periods in terms of the total number of births for each year, mode of delivery and booked/un-booked participants for all three hospitals using MedCalc’s odds ratio calculator (www.medcalc.org/calc/odds_ratio.php). Statistical significance was accepted when the *P*-value was < 0.05.

### Ethical approval and consent to participate

Approvals and permissions were obtained from the authority of the study hospitals. The study was approved by the Ethics Review Board of NAUTH, Nnewi, Nigeria (reference number: NAUTH/CS/66/VOL.13/VER III/46/2020/038 26–2017; date of approval: 31st July, 2020) and the other two hospitals (Reference numbers: UNTH/CSA/329/VOL.5; ESUTHP/C-MAC/RA/034/VOL.1/293). The study was conducted according to the Helsinki declarations on ethical principles for medical research involving human subjects. The collected data were kept confidential and accessed only by the research team members.

## Results

**Figure 1:**
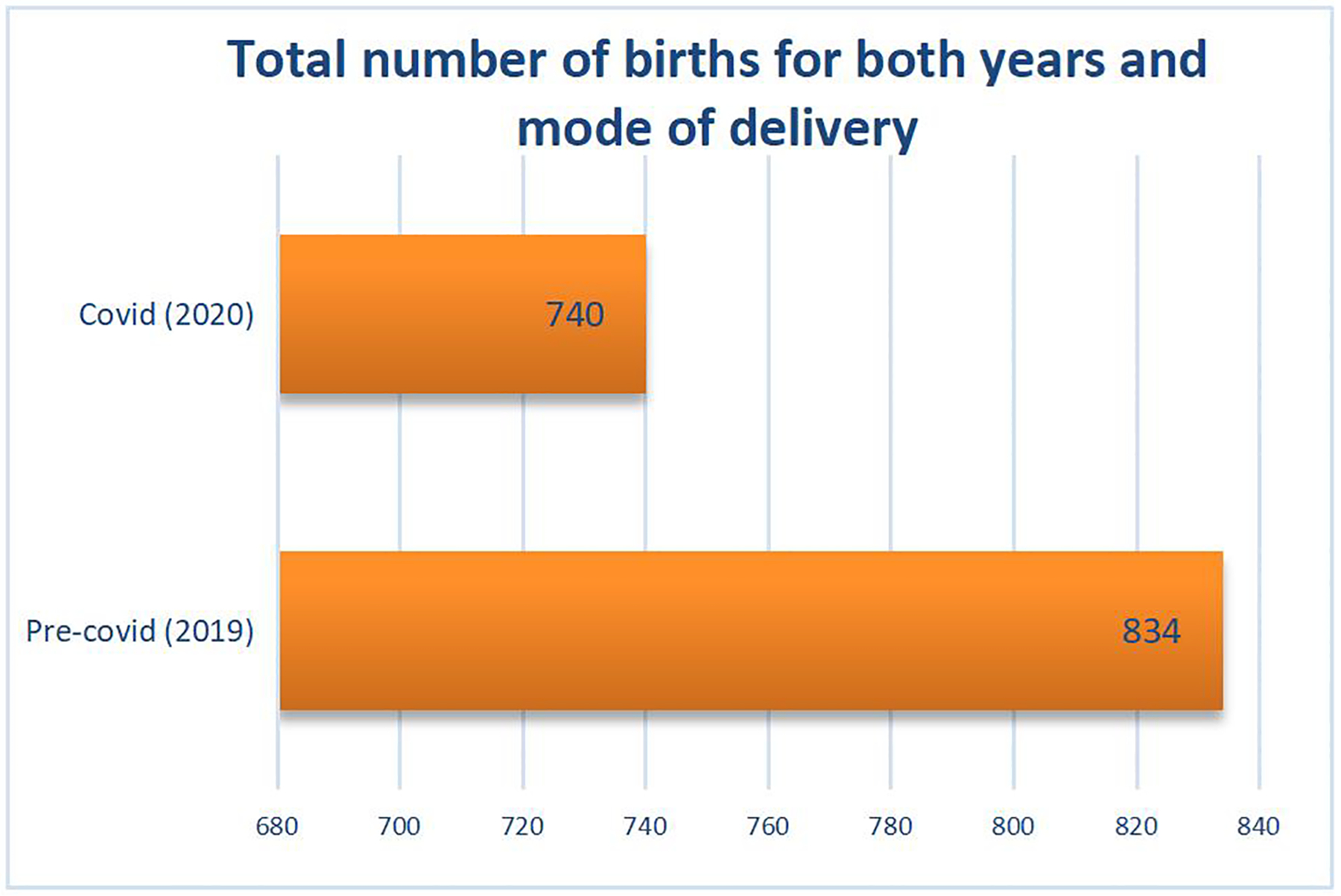
Total number of births during the study period

Overall, the total number of births for all three hospitals in 2019 was 3,184 and 2,544 in 2020. However, the total number of births for all three hospitals for the 3 months of (October to December) 2019 was 834 (52.9%) and 740 (47.1%) for 2020, a difference of 94 births or 11.3% decrease in birth rates from the pre-pandemic. This is shown in Figure 1. There was a significance statistically (*P* = 0.0009) with a 95% CI of −16.0519 to −4.1481. The standard deviation for the births in 2019 was 59.26 and that of 2020 was 60.86. The 834 and 740 births number reported in 2019 and 2020 represent 26.19% and 29.09% of births studied respectively.

In addition, the total number of vaginal delivery during the pre-COVID (2019) period was 444 (53.2%), while that of 2020 was 442 (59.7%) as seen in Table 1. Whereas for cesarean section the total number was 390 (46.8%) for 2019 and 298 (40.3%) for the same months in 2020. The odds ratio of the vaginal mode of delivery compared to cesarean section (CS) for the hospitals was 1.3028 with a 95% confidence interval of 1.0664 to 1. 5916. There was a significant difference (*P* = 0.0096) in the mode of delivery in both years.

**Figure 2:**
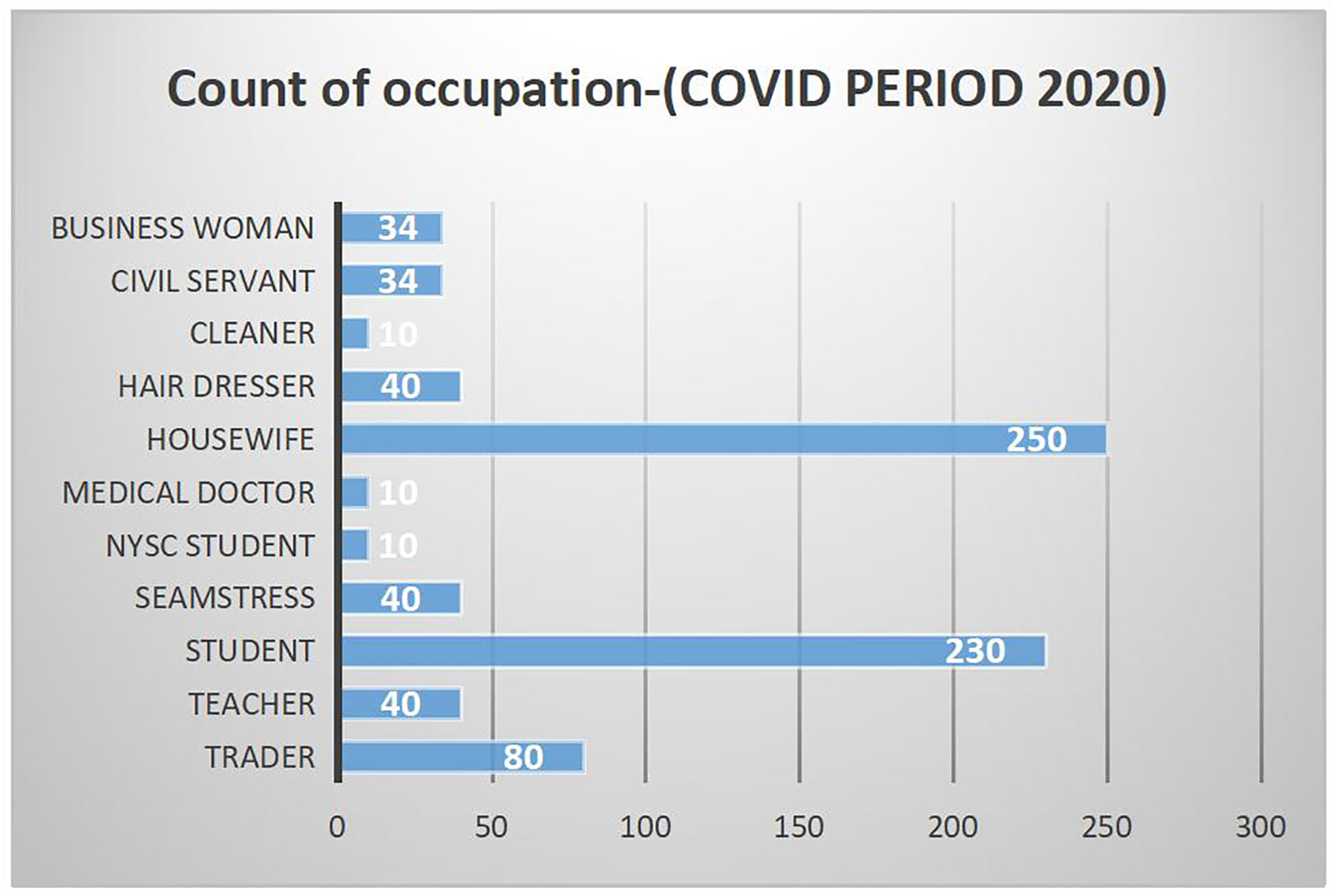
The occupation of participants. NYSC, National Youth

**Figure 3:**
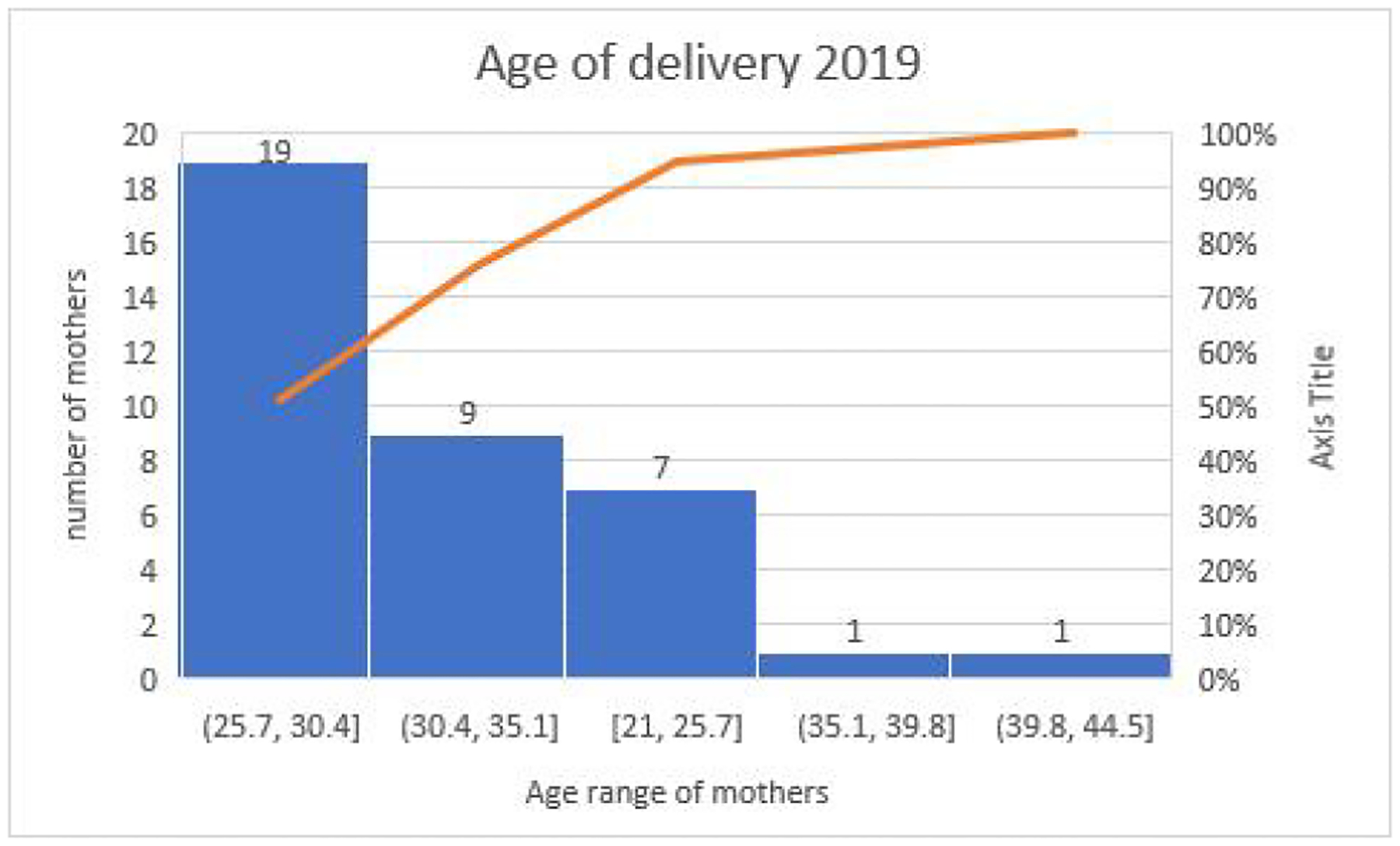
Age of delivery during the pre-pandemic

**Figure 4:**
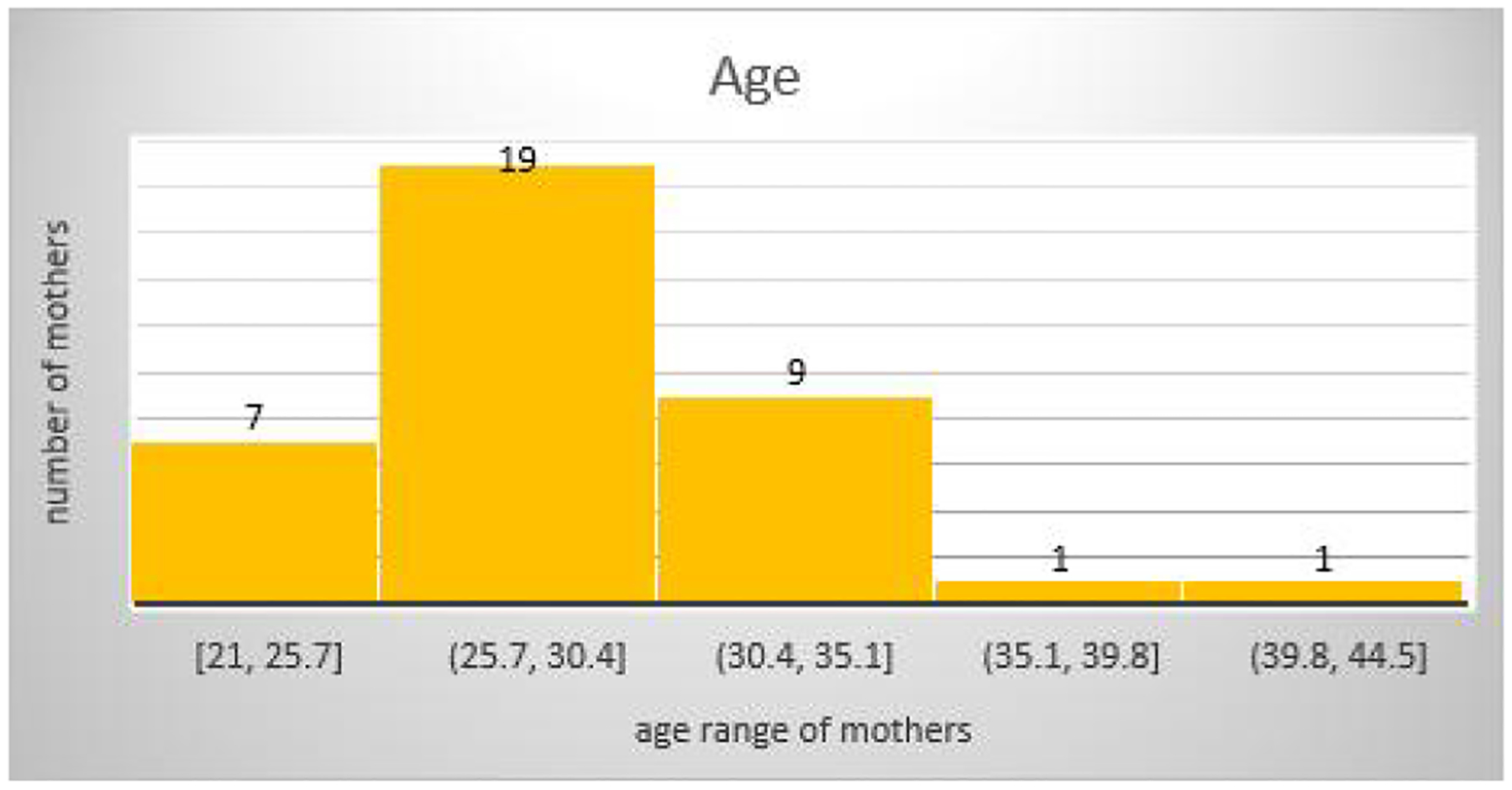
Age of delivery during the pandemic

The percentage of booked participants for 2019 was 79.4% compared to 77.7% for 2020, while that of un-booked participants was 20.5% for 2019 and 22.3% for 2020. The percentage of booked patients fell by 1.7% and that of un-booked participants rose by 1.8% in the COVID period as seen in Table 2. There is no significant difference (*P* = 0.3937), the 95% CI is 0.7081 to 1.1455 and the odds ratio was 0.9006 for the occupational demography among the mothers. In 2020 (Figure 2), Students and Housewives gave birth more compared to Medical Doctors and Cleaners who had the least numbers whereas. In 2019 more women who were Teachers and Traders gave birth as compared to Students, Hairdressers and Computer Operators whose numbers were the lowest. The age demographic of mothers was highest within the 25–30 age group followed closely by 30–35 in the year 2019 (Figure 3) within study months. This finding was similar for 2020 (Figure 4).

## Discussion

Our study shows that there was a decline in the tertiary hospital-based number of deliveries in all three hospitals of study during the 3-months (October-December) COVID-19 period, from 833 in 2019 to 742 in 2020, a difference of 94 births, which was significant statistically (*P* = 0.0009). This illustrate an 11.3% decrease in birth rates when compared with the pre-pandemic period. A similar decrease was reported in Mozambique which reported a birth decrease of 4% during the pandemic period [7]. Similarly, in a recent review by Senkyire et al., on the impact of the COVID-19 pandemic on maternal and child health in Africa, it was reported that COVID-19 was associated with reduced facility-based births in Africa [8]. Conversely, a French study by Quibel et al. did not reveal any significant decrease in the birth rates during the two periods [9].

It is important to explore the possible causes of this finding. The majority of this reduction may not only be due to the impact of the pandemic and lock-down measures themselves as the psycho-social indices of unemployment and economic recession in the country before the pandemic which worsened during the COVID period may play an important role in this trend. For example, the exchange rates between naira and dollar saw an all-time increase to 495 per 1 dollar before the COVID period, causing the increase in the prices of imported goods and raising the cost of living for most people invariably affecting their mental health and fertility [10]. The reduction in birth rate itself shows that there is a declining number of tertiary hospital-based deliveries among mothers. Millions of people were unable to work. However, this latter hypothesis was most probable reason according to our results.

The lockdown caused a lot of people not to be able to go about their regular jobs which led to financial constraints and difficulties, such situations have been postulated to induce psychological stress which invariably affects fertility in the form of premature ejaculations and loss of libido, so there is a direct relationship between poverty, economic recession and fertility [1, 10]. Hence the government and other financial bodies need to have a dialogue on how to salvage the economic crisis as this may be having an impact on the reproductive life of their citizens.

Furthermore, other key factors could serve as players in this trend. To begin with, the pandemic came with developments such as total lockdowns and curfews, this led to restriction of movements for most patients, including pregnant women and may have led to them delivering at home or other places more convenient for them. It is possible that these women could not access care at any hospital due to the total lockdown and possibly had complications, lost their babies or even died.

Secondly, tertiary hospitals had to modify their operations as some became isolation centres for COVID-19 patients. Delivery represents a unique situation in the COVID-19 pandemic, and all hospital admissions and multiple admissions are expected to be planned based on the new set of guidelines by these tertiary hospitals because of the pandemic [11]. In anticipation of hospital admission and to limit the hazard of exposure, women are frequently recommended to discontinue work or start working from home a minimum of two weeks before the expected date of delivery and to exercise strict social isolation throughout this time, for most females this must be initiated at 37 weeks of gestation [11]. These protocols in addition to the COVID-19 isolation centres present in these hospitals may have made women reluctant and scared to use the tertiary hospitals for delivery as it might put them at risk of being exposed to the COVID-19 infection and affect their jobs/lifestyles.

Also, some women stayed away from tertiary hospitals for fear of being labeled COVID-19 patients whether rightly or wrongly so, because of the level of stigmatization that was associated with the condition during that period. More factors that may have caused this decline include; disruptions in hospital visits for assisted reproduction and increased mortality from the pandemic. Hence, there is, therefore, an urgent need for organizational changes to healthcare delivery to protect those vulnerable to the virus (staff and patients); to protect them from potentially harmful consequences such as being infected with the virus or psycho-social problems like phobia and stigmatization.

Pregnant women, in particular, are encouraged to take all available precautions to optimize health [9]. Avoid exposure to COVID-19 and continue with their antenatal care in these hospitals. Additionally, some studies extrapolated that there is a dynamic complex situation between COVID-19 pandemic and fertility which depends on individual behaviors, and country of residence. Therefore, the researchers proposed some working theories which were: 1) Rise in fertility from the lockdown due to close range between spouses, decrease in the use of contraceptives and increased in adolescent fertility. 2) Fallen fertility as a result of increased conflicts and divorce, fear and economic hardship, 3) No effect on fertility [10, 12–14].

Our multi-centre analysis depicted that there was a significant change in the mode of deliveries (*P* = 0.0096) as more mothers gave birth through vaginal delivery 59.7% compared to 40.3% during the post-lockdown months. A similar study done recently showed that despite the fact emergency cesarean section was unaffected, there was an overall significant fall in the number of elective cesarean sections during the lockdown period [15]. This could be due to fewer women having access to healthcare during the lockdowns or phobia of hospitals during that period. On the other hand, another recent study reviewed the mode of delivery during COVID-19 between December 2019 and April 2020 and found that 68.9% of women gave birth via CS with COVID status alone being a common indicator [16].

Further research will be recommended to throw more light on this prominent observation. The percentage of booked patients fell by 1.7% and that of un-booked patients rose by 1.8% in the COVID-19 period, this finding was not statistically significant. The age range of delivery showed that women within the age range of 25–35 had the highest rate of delivery in all hospitals for the study period. Which is usually the peak period of fecundity for most women the world over. There is scarce literature on birth rates during the COVID-19 era in Nigeria, hence our study builds a foundation from which many other works can spur and shows a significant impact of the pandemic in Nigeria. The sample size obtained from the three tertiary hospitals spread across the South-Eastern part of Nigeria is a strength of our multi-centre study, making our findings relatable to most public health-care settings with frequent deliveries in Nigeria. This study however does not account for deliveries in private sectors and does not explore some other socio-demographic parameters. Also, we only studied for a 3-month period, each of pre and during the pandemic. It would have been more comprehensive if the period of the study could be longer in order to get more information and have more impact, since the pandemic lasted two years and the study just a few months.

### Significance statement

This study discovered that there was not only a reduction in the birth rate among mothers who visited Tertiary hospitals but there was a decline in the rate of cesarean section, this may be a transient situation as seen with other pandemics, however, this finding can be beneficial for uncovering ways pregnant women can have more access to quality health care during natural disasters and pandemics. This study will help the researchers to uncover the critical areas of fertility and how key factors such as community stigma of an illness, economic hardship and poor hospital and government policies can affect the birth rate during a Pandemic and further studies can be done to help clarify some grey areas such as a decrease in cesarean section and COVID-19 as witnessed this period.

## Conclusion

In conclusion, the birth rate among the three tertiary hospitals in Nigeria showed that there was a reduction in tertiary-hospital based birth rate during the COVID-19 period compared to the pre-COVID period, which was statistically significant. Our study highlighted factors related to the COVID-19 restrictions such as decreased access to healthcare, phobia of hospitals, stigmatization from visits, growing economic recession, inflation and increased unemployment rates that may have played a role in the decline in birthrates.

## Figures and Tables

**Table 1:** Mode of delivery and total number of births

Mode of delivery	Pre-covid (2019)	Covid (2020)
Vagina	444 (53.24%)	442 (59.73%)
Caesarean section	390 (46.76%)	298 (40.27%)
Total	834	740

**Table 2:** Total of booked and unbooked patients

	Pre-Covid (2019)	Covid (2020)
Booked	654 (79.4%)	589 (77.7%)
Unbooked	169 (20.5%)	169 (22.3%
Total	823	788

## Abbreviations

COVID-19: coronavirus disease 2019
ESUTH: Enugu State University Teaching Hospital
NAUTH: Nnamdi Azikiwe University Teaching Hospital
CS: cesarean section
UNTH: the University of Nigeria Teaching Hospital
